# Synthesis and Characterization of a Trifunctional Photoinitiator Based on Two Commercial Photoinitiators with α-Hydroxyl Ketone Structure

**DOI:** 10.3390/ma14185272

**Published:** 2021-09-13

**Authors:** Rong Zhong, Hui Hu, Yanfang Zhou

**Affiliations:** Department of Material Chemistry, School of Environment & Chemical Engineering, Nanchang Hangkong University, Nanchang 330063, China; 1902081704113@stu.nchu.edu.cn (H.H.); 2019620052@gzhmu.edu.cn (Y.Z.)

**Keywords:** trifunctional photoinitiator, hydroxyl ketone, synthesis, migration, photopolymerization

## Abstract

A trifunctional photoinitiator based on commercial photoinitiators 2-hydroxy-2-methylpropiophenone (Irgacure1173) and 2-hydroxy-4′-(2-hydroxyethoxy)-2-methylpropiophenone (Irgacure2959) was synthesized by an esterization reaction. Its structure was characterized by UV-Vis spectrometry, Infrared Transformed Fourier, Proton Nuclear Magnetic Resonance Spectra, 13 Carbon Nuclear Magnetic Resonance Spectra, Mass Spectrometry, and Thermogravimetry. In addition, its photoinitiating activity was investigated. The results showed that the novel photoinitiator had good photoinitiating activity and thermal stability compared to commercial photoinitiators. The migration of the residual photoinitiator in the cured film was lower than that of 1173 and 2959.

## 1. Introduction

UV curing technology has been developed quickly due to its high efficiency, environmental friendliness, energy savings, and good economics [1,2,3]. UV-cured materials have been used in coatings [4,5], adhesives [6], inks [7], electricity [8], biomaterials [9], 3D printing [10], and so on. Usually, UV systems consist of photoinitiators, oligomers, reactive diluents, and other additives. Photoinitiators, as an important component of the UV system, are small organic compounds that can break down into smaller compounds. The available types of photoinitiators include free radical photoinitiators [11,12,13], cation photoinitiators [14,15], and anion photoinitiators [16,17]. Currently, free radical photoinitiators are predominant.

2-Phenyl-acetophenone derivatives are well-known free radical photoinitiators that can generate two small radicals via α-cleavage after UV irradiation [18,19,20]. On the one hand, free radicals can initiate the polymerization of double bonds. On the other hand, residual radicals or photoinitiators can induce yellowing and migration in the system, which can affect the properties of UV-cured materials. For this reason, the current trend of UV-curing formulations is towards the development of photoinitiators that simultaneously have high activity, less yellowing, and relatively low migration [21,22,23,24].

To overcome these disadvantages of yellowing and migration, some larger molecular weight photoinitiators have been explored [25,26,27,28,29,30]. Some researchers have prepared two or more functional photoinitiators for improving some properties of UV cured films [31,32].

In our past work, a tetrafunctional photoinitiator with the same initiating group tetraphenoylformate were developed [33]. In this paper, a trifunctional photoinitiator based on two different photoinitiators was synthesized by esterization. The structure of the trifunctional photoinitiator was characterized by UV, Infrared Transformed Fourier, Proton Nuclear Magnetic Resonance Spectra, 13 Carbon Nuclear Magnetic Resonance Spectra, Mass Spectrometry, and Thermogravimetry. The initiating activity and migration of the new photoinitiator were investigated. The property of chemical resistance of the cured films was investigated, like acid and base resistances. The new photoinitiator has potential applications for food and drug packages.

## 2. Experimental

### 2.1. Materials

B-265, difunctional polyurethane acrylate, and reactive diluent, 1,6-hexanediol diacrylate, HDDA (>98%), trimethylolpropane triacrylate TMPTA (>98%) were all industry grade and were bought from Guangdong Boxing New Material Technology Co., Ltd., Guangzhou, China. The other chemicals were analytical grade and were provided by Shanghai Aladdin Biochemical Technology Co. Ltd., Shanghai, China. The Irgacure 1173 and Irgacure 2959 provided by BASF (Ludwigshafen, Germany)that were used were analytical grade and used without further purification.

### 2.2. Preparation of the Trifunctional Photoinitiator (HTH)

**Figure 1:**
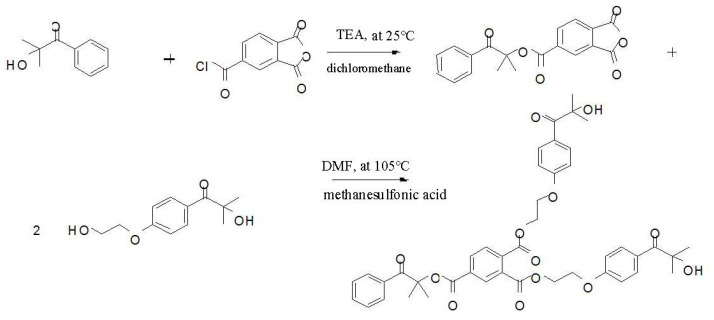
Synthesis route of HTH.

The synthetic route of trifunctional photoinitiatorswere is shown in Figure 1. It was prepared by the following procedure. Photoinitiator 1173 3.28 g (0.02 mol) and triethylamine 2.25 g (0.022 mol) were added into a three-necked flask equipped with a reflux condenser and then dissolve ed in 50 mL dichloromethane by magnetic stirring at room temperature. The reaction was carried out at room temperature, and trimellitic anhydride chloride was dissolved in 20 mL dichloromethane, added into a constant pressure drop funnel, and slowly dripped into the three-necked flask. The stirring reaction was continued overnight, filtered to remove the triethylamine hydrochloride, washed 3 times with 50 mL deionized water each time, and put into a vacuum desiccator to dry at 70 °C for 6 h. The obtained organic phase was purified by column chromatography. The developing agent of the silica gel column was a mixture of petroleum ether and ethyl acetate. The specific ratio of V (petroleum ether)–V (ethyl acetate) = 2:1. The product obtained was a yellowish liquid.

The products above 3.38 g (0.02mol), 2959 photoinitiators4.48 g (0.02mol), dimethylformamide (DMF) and methanesulfonic acid, were added into a three-necked flask. The reaction was started at 105 °C under the protection of stirring and nitrogen. The reaction was performed at 105 °C for 4 h and then cooled. Next, 40 mL dichloromethane was added to dissolve the product, followed by washing with 50 mL saturated salt water, washing twice with 50 mL deionized water, and drying in a vacuum desiccator at 70 °C for 6 h. The collected organic phase was further purified by column chromatography. The developing agent in the silica gel column was a mixture of petroleum ether and ethyl acetate with a ratio of V (petroleum ether)–V (ethyl acetate) = 2:1, and a yellow substance4.86g was obtained and named Benzene-1,2,4-tricarboxylic acid 4-(1,1-dimethyl-2-oxo-2-phenyl-ethyl)eter1,2-bis-{2-[4-(2-hydroxy-2-methyl-propionyl)-phenoxy]-ethyl} ester, HTH, with a yield of 63.2%.

### 2.3. Preparation of UV Curable Films

First, 60%, 36%, and 4%, respectively, of b-265 polyurethane resin, HDDA monomer, and photoinitiator were put into a beaker, stirring evenly for 30 min. The thickness of the films was approximately 30 μm. The films were cured in air by a hand-held curing machine. The curing machine parameters were set as a lamp spacing of 5.5 cm and a power of 400 W. A high-pressure mercury lamp was chosen as the light source in the experiment, and its main wavelength was 365 nm.

### 2.4. Characterization of the Products

A Fourier infrared spectrometer (KBr tablet, Nicolet210, Waltham, MA, USA), T6 ultraviolet spectrometer (acetonitrile solvent, Beijing Pu Analysis General Instrument Co., Ltd., Beijing, China), Bruker 400 MHz NMR spectrometer (CDCl_3_ solvent, TMS internal standard, 25 °C, Bruker, Karlsruhem BW, Germany), Mass spectrometer LCQ DECA XP (acetonitrile solvent, electrospray dissociation source, Thermo, Waltham, MA, USA),TG-209 Thermogravimetric Analyzer (Netzsch, Selb, Germany), photodifferential scanning calorimeter (Perkin Elmer, Waltham, MA, USA), radiometer AS813 (Smartsensorinstruments Co. Ltd., Dongguan, China and UVA-T-400W portable UV curing light (Dongguan Gu Dejia Machinery Equipment Co., Ltd., Dongguan, China) were used.

The surface dry time of the coating was tested by the finger touch method. The pencil hardness test was conducted according to the ASTM D3363-05 standard. The adhesion refers to ASTMD3002 (0B-5B).Testing acid and base resistances was conducted putting the sample into the acid (0.1 mol·L^−1^ HCl) or base (0.1 mol·L^−1^ NaOH)solution for 24 h at room temperature.

### 2.5. Migration of the Photoinitiators

A TMPTA solution of 1 (*w*/*w*) % photoinitiator (1173, 2959, HTH) was prepared. Each solution was coated on a glass slide with a thickness of approximately 30 μm. The films were exposed to air in a portable curing machine with a lamp width of 5.5 cm and a power of 400 W for 3 min. The cured film was then cut, with each piece weighing 0.075 g, and extracted with 20 mL acetonitrile at room temperature for 3 days. Finally, equal amounts of extract were sampled for the UV absorption test. The relative mobility R of HTH relative to the 1173 photoinitiator and the 2959 photoinitiator can be obtained from Equations (1)–(3):

c = A/(ε × b)(1)

R_1_ = c (HTH)/c (1173) (2)

R_2_ = c (HTH)/c (2959) (3)

In these formulas, c is the concentration of the photoinitiator in the extract, mol/L; A is the absorbance; ε is the molar extinction coefficient, L/(mol·cm); b is the sample cell thickness, cm; c (HTH) is the concentration of HTH in the extract; c (1173) is the concentration of the 1173 photoinitiator in the extract; c (2959) is the concentration of the 2959 photoinitiator in the extract; and R is the relative mobility of HTH.

### 2.6. Photoinitiator Activity

The photo-DSC experiments were carried out with Pekin Elmer DSC800 equipped with a UV curing system (OmniCure Series 2000, Excelitas Technologies Corp., Waltham, MA, USA). The samples (about 1 mg) were placed in an aluminum pan andcovered quartz disc. Heat flow versus time curves were recorded in isothermal mode under nitrogen flow (200 mL/min). The theoretical heat for complete acrylic double bond conversion is 86 kJ·mol^−1^. The lamp intensity is 10 W/cm^2^.

## 3. Results and Discussion

### 3.1. FTIR

**Figure 2:**
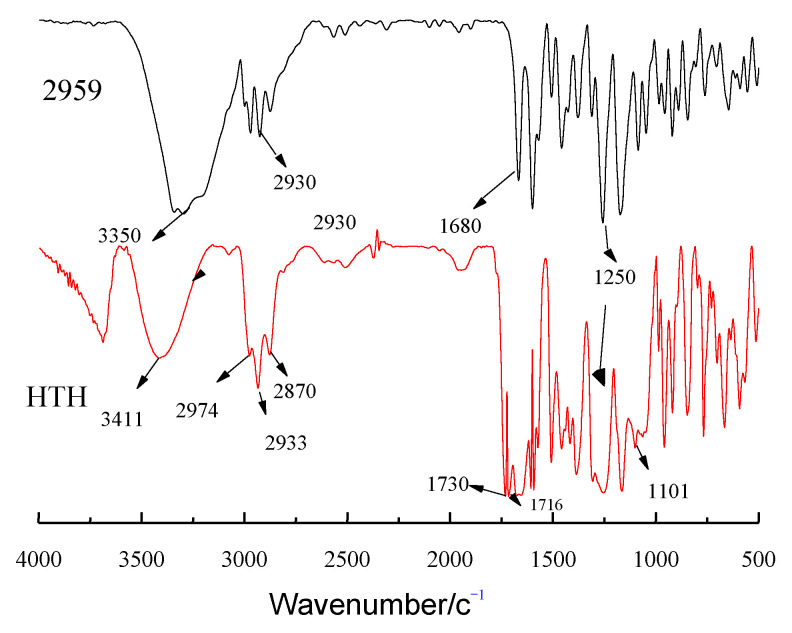
FTIR spectra for HTH and 2959.

Figure 2 compares the FTIR spectra of 2959 and the HTH product. The great weakening of the broad band at approximately 3450–3500 cm^−1^ is attributed to -OH stretching with hydrogen bonds. The peaks at 2974 cm^−1^ and 2870 cm^−1^ are the anti- and symmetric stretching vibration absorption peaks of -CH_2_, and those at 2933 cm^−1^ are asymmetric stretching vibration absorption peaks of -CH_3_. The absorption peaks locating at 1690–1680 cm^−1^ is stretching vibrations of aromatic ketones. The stretching vibrations of C-O-C is about 1200 cm^−1^. For HTH, the vibration absorption peak of C=O in the saturated ester carbonyl group is at 1710–1750 cm^−1^. Because the HTH molecule contains three ester groups, the electric dipole moment of the ester carbonyl is large, and the absorption is strong and broad.

### 3.2. NMR

**Figure 3:**
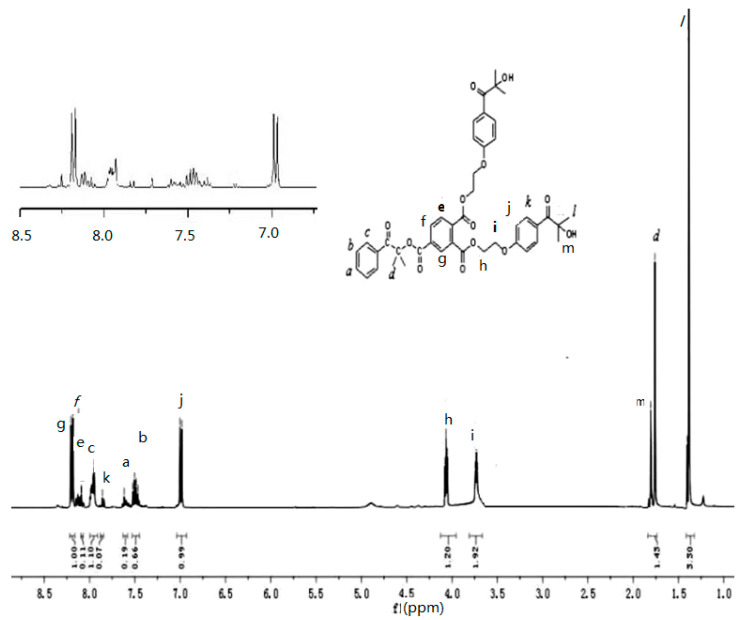
^1^H NMR spectrum of HTH.

**Figure 4:**
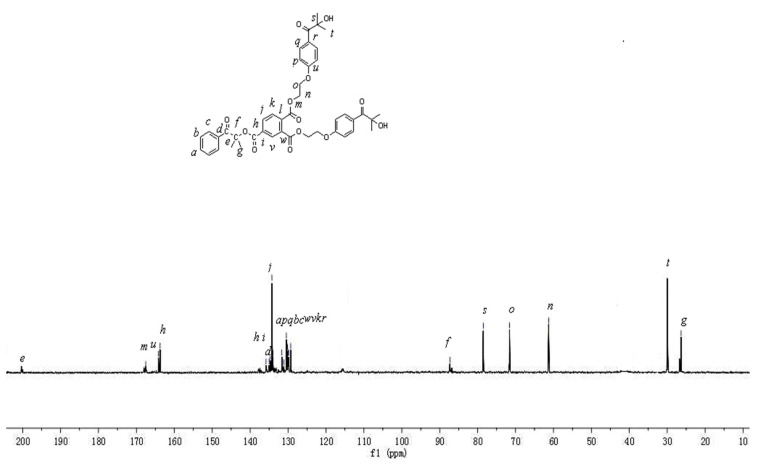
^13^C NMR spectrum of HTH.

^1^H NMR and ^13^C NMR spectra of the HTH in CDCl_3_ are shown in Figure 3 and Figure 4, respectively. The data are as follows: ^1^H NMR (400 MHz, CDCl_3_) δ 8.19–8.21 (d, J = 9.0 Hz, 1H), 8.08–8.09 (d, J = 1.7 Hz, 1H), 8.00–7.91 (m, 1H), 7.86 (s, 1H), 7.62 (s, 1H), 7.47–7.49 (dd, J = 14.7, 7.2 Hz, 1H), 6.98–7.00 (d, J = 9.0 Hz, 1H), 4.05–4.08 (t, J = 4.9 Hz, 1H), 3.72–3.74 (t, J = 4.9 Hz, 2H), 1.76 (s, 1H), 1.39–1.40 (d, J = 7.0 Hz, 3H). ^13^C NMR (101 MHz, CDCl_3_) δ 167.54 (s), 164.17 (s), 163.75 (s), 135.81 (s), 135.00 (s), 134.68 (s), 134.28 (s), 134.14 (d, J = 23.4 Hz), 131.92 (m), 131.05 (m), 130.44 (s), 130.36 (m), 129.29 (s), 87.32 (s), 78.52 (s), 71.59 (s), 61.28 (s), 29.84 (s), 26.34 (s).

The signals at 167.54 ppm, 164.17 ppm, and 163.75 ppm in the ^13^C NMR spectra of the HTH are attributed to the carbon atoms of the three carbonyl groups, confirming the existence of ester bonds in the HTH. The signals of the hydrogen and carbon atoms in the ^1^H NMR and ^13^C NMR were basically consistent; thus, it can be inferred that the HTH multifunctional macromolecular photoinitiator was successfully synthesized.

### 3.3. MS

**Figure 5:**
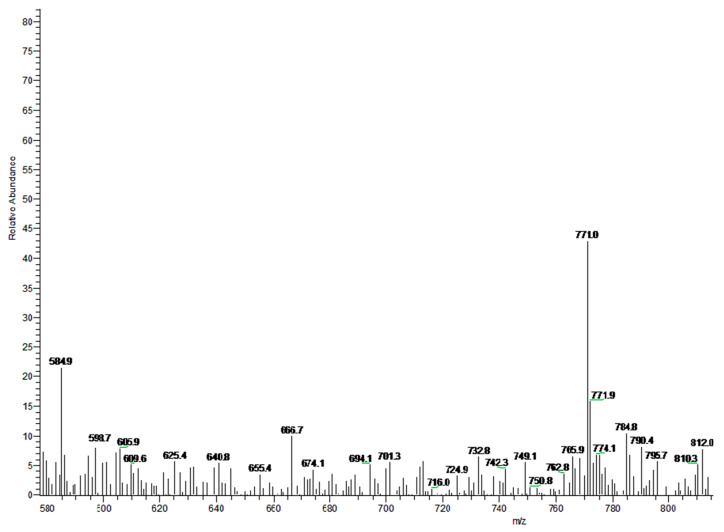
MSI-MS spectrum of HTH.

Figure 5 presents the MSI-MS spectrum of the HTH. The molecular weight is 771.0, and the peak at 771.9 is the M + 1 peak of the HTH, which is consistent with the relative molecular weight. Because the deposition of HTH produces many fragments, there are a lot of peaks in the MSI-MS. The strongest peak is consistent with the photoinitiator HTH.

### 3.4. UV Absorption Spectra

**Figure 6:**
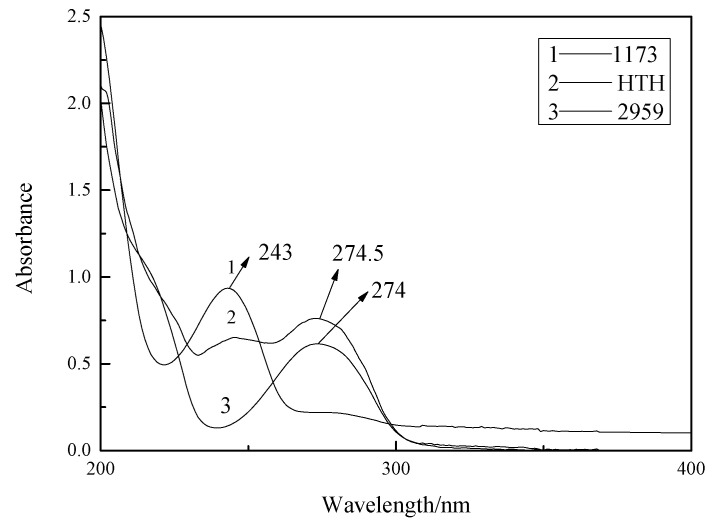
UV-Vis spectra of 1173, 2959, and HTH (solvent: acetonitrile, 25 °C).

Figure 6 shows that the maximum absorption peaks of 1173 and 2959 for the HTH photoinitiator are at 243 nm, 274nm, and 274.5 nm, respectively. Table 1 shows the maximum molar absorption coefficient related to the maximum absorption wavelengths of the 1173, 2959, and HTH. Therefore, the maximum UV absorption wavelengths of the HTH and the 2959 photoinitiator are very similar. Compared with the 1173 photoinitiator, the HTH has a higher maximum UV absorption wavelength because the molecular configuration of the HTH contains more chromophiles, such as C=O (C=O * excited transitions). In addition, due to the power supply from the phenoxy groups, the integral area of the HTH photoinitiator in the 245–300 nm band is larger than that of the 1173 photoinitiator. It corresponds to the small molecule 1173 photoinitiator and thus improves the photosensitive efficiency of the UV curing system in the long wavelength region.

### 3.5. Thermogravimetric Analysis (TGA)

A TG-209 thermogravimetric analyzer was selected to analyze the thermal stability of the 1173, 2959 and HTH photoinitiators. The heating scope of the equipment is generally set as 70–500 °C, the heating rate is 10 °C/min, the temperature is maintained for 20 min, the data are collected every 0.5 s, and the atmosphere is nitrogen.

**Figure 7:**
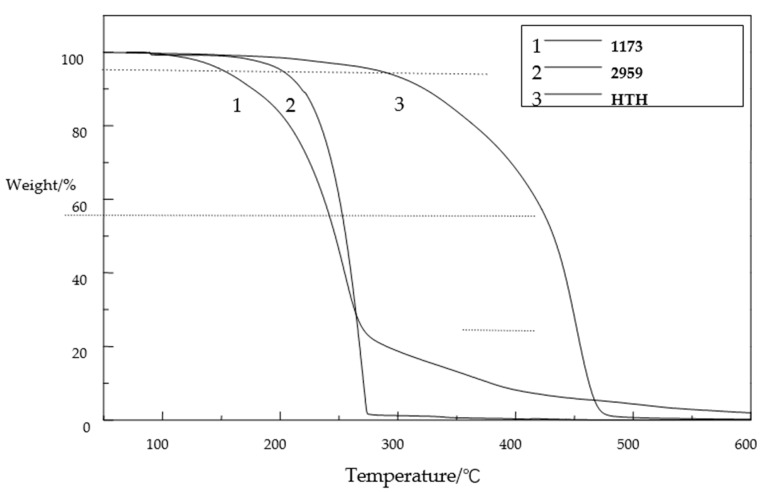
TG spectrum of 1173, 2959, and HTH.

From Figure 7, it can be seen that the thermal degradation process can be roughly divided into three stages: First, 70–290 °C for phase 1. The reduction of HTH is not obvious, and any loss in general is because of the HTH curing system due to moisture, part of itself, and water inhibition being volatile due to the free type of these small molecules. The second stage is 290–437 °C. In this stage, the HTH begins to decompose into small molecular substances, which is the main weightlessness stage. For the third stage, it is 437–490 °C, in which the HTH is cracked and the weight loss rate tends toward zero until the cracking of HTH is completed. Therefore, HTH shows good thermal stability and nonvolatilization, which extends its storage life. The temperature of the HTH photoinitiator increased by 5%, the temperature difference between the HTH photoinitiator and the 2959 photoinitiator increased by more than half, and the temperature difference of the 70% weight loss between the HTH photoinitiator and the 2959 photoinitiator was small, so the thermal stability of the HTH photoinitiator was slightly better than that of the 1173 and 2959 photoinitiators.

### 3.6. Photopolymerization Kinetics Analysis

**Figure 8:**
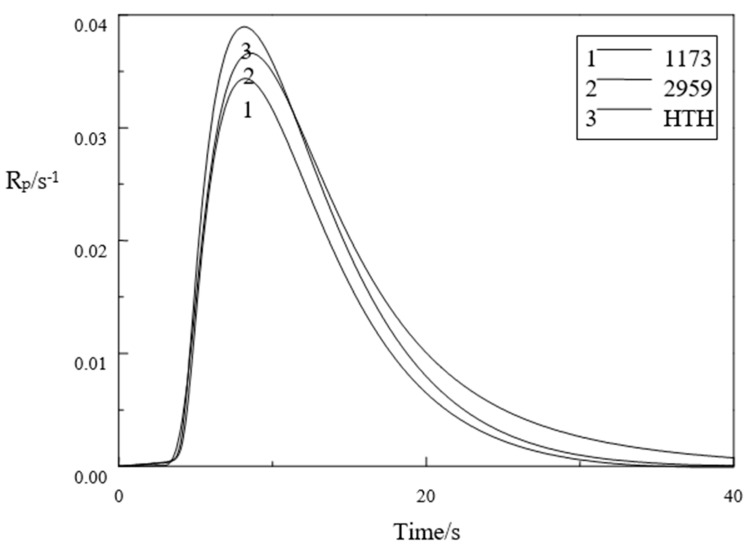
Curves of 1173, 2959, and HTH-initiated polymerization reaction rate of TMPTA.

The polymerization reaction rate and conversion rate over time are shown in Figure 8. At 25 °C in a nitrogen environment, surrounded by an empty aluminum crucible as a comparative sample, the light intensity was set to 10 mW/cm^2^, illumination 100 s, photoinitiator concentration 0.006 mol, and monomer 2 g TMPTA.

**Figure 9:**
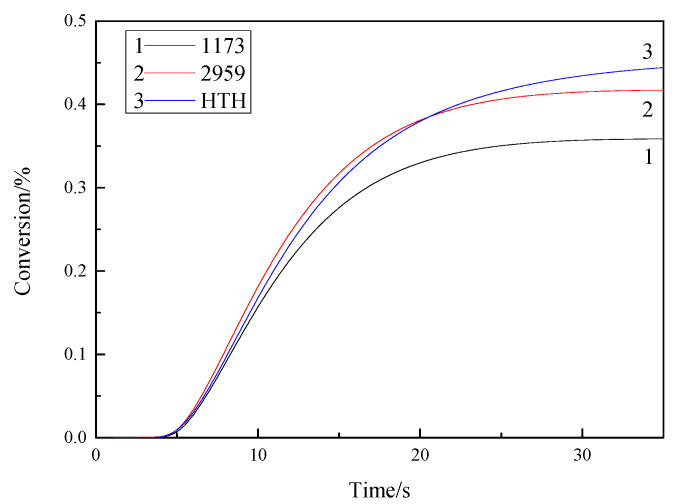
Curves of 1173, 2959, and HTH-initiated polymerization conversion rate of TMPTA.

Figure 8 and Figure 9 show that the photoinitiator activity of HTH was higher than that of the 1173 photoinitiator. The reason for this is that the concentration of active free radicals formed after HTH photolysis exceeded the 1173 photoinitiators, which in turn ledto faster TMPTA polymerization by HTH. After 12 s, the reaction rate when HTH initiated TMPTA polymerization gradually increased relative to that of the 2959 photoinitiator, and after 20 s, the double bond conversion rate when HTH initiated TMPTA polymerization gradually increased relative to that of the 2959 photoinitiator. This is generally because the local free group concentration of HTH is large, which can more effectively inhibit oxygen resistance and increase photoinitiator activity. The monomer double bond conversions DBC (%) for the different photoinitiator systems 1173, 2959, and HTH are 35%, 40%, and 42%, respectively. The rate of polymerization Rp (mmol/L/s) of 1173, 2959, and HTH systems are 0.034 s^−1^, 0.036 s^−1^, and 0.039 s^−1^.

### 3.7. Properties of the Cured Films

Table 2 shows that the cured film initiating HTH has high hardness and excellent adhesion and can be applied in various acidic and alkaline conditions. It is also compatible with resin.

### 3.8. Migration of Residual Photoinitiator in the Cured Films

**Figure 10:**
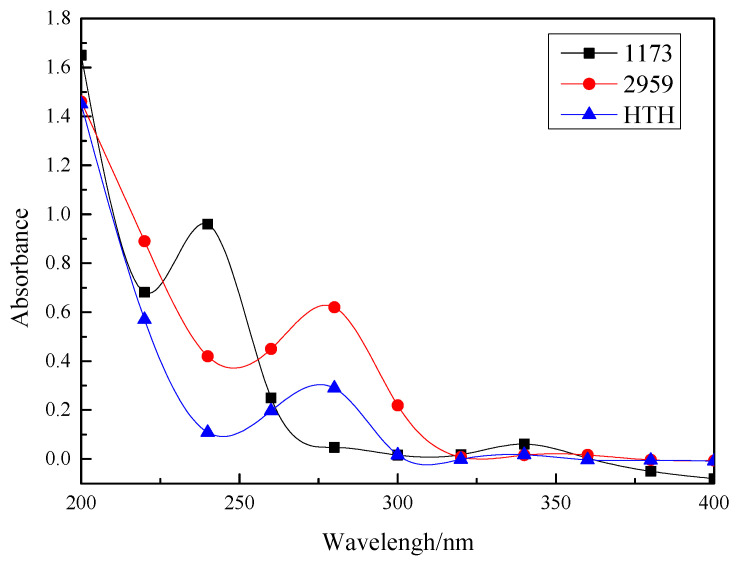
UV-Vis absorption spectra of extraction solutions from 1173, 2959, and HTH.

The UV absorption of the photoinitiator extract in the polymer can explore the migration capacity of the photoinitiator in the polymer, that is, the residual amount of the photoinitiator in the solution can be obtained through the correlation of the absorbance and the concentration at various wavelengths. The UV absorption spectra of the 1173, 2959, and HTH photoinitiator extracts are shown in Figure 10. Table 3 show the relative mobility R of the 1173, 2959, and HTH photoinitiators.

The absorption peaks at 1173, 2959, and HTH remained at the maximum wavelength, confirming that the curing membrane remained a light initiator. The HTH system migration rate was only 41.80% of the 2959 light initiator system and only 20.65% of the 1173 light initiator system. This also confirmed that the trifunctional HTH photoinitiator is more efficient in curing systems, which in turn reduces the toxicity of the cured film.

## 4. Conclusions

A novel trifunctional photoinitiator HTH was successfully synthesized on the basis of the commercial photoinitiators 1173 and 2959 hydroxyl ketones, and its structure was characterized. The new photoinitiator has good thermal stability and initiating activity, better than 1173 and 2959. The migration of the residual photoinitiator of the cured film after UV radiation was obviously lower than that of 1173 and 2959. It has good potential for application in food and drug packages in the UV curing field.

## Figures and Tables

**Table 1:** The maximum absorption wavelength and maximum molar extinction coefficient of 1173, 2959, and HTH.

Photoinitiators	1173	2959	HTH
λ_max_/nm	243	274	274.5
ε_max_/(×10^4^ L·mol^−1^·cm^−1^)	9.2	5.7	7.8

c/(×10^−5^ mol·L^−1^).

**Table 2:** Film properties.

Photoinitiator	1173	2959	HTH
hardness/H	3	4	4
adhesion	2	2	3
ductility	good	good	good
Acid resistance *	No crack	No crack	No crack
Base resistance **	No crack	No crack	No crack

* 0.1 mol·L^−1^ HCl, ** 0.1 mol·L^−1^ NaOH (for 24 h).

**Table 3:** The migration of HTH, 2959 relative to 1173.

Photoinitiator	HTH	2959	1173
A(λmax)	0.32	0.605	0.96
c/(mol·L^−1^)	3.47 × 10^−6^	8.3 × 10^−5^	1.68 × 10^−5^
R/%	20.65	49.40	100

## Data Availability

Data sharing is not applicable.
